# Editorial: Protein Functional Changes and Signaling Transduction in Cancer Stem Cells

**DOI:** 10.3389/fonc.2021.761678

**Published:** 2021-09-15

**Authors:** Shihori Tanabe

**Affiliations:** Division of Risk Assessment, Center for Biological Safety and Research, National Institute of Health Sciences, Kawasaki, Japan

**Keywords:** cancer stem cell (CSC), molecular network, protein function, signaling pathway, signaling transduction

## Phosphorylation of Endothelin-Converting Enzyme-1c at Serines 18 and 20 by CK2 Promotes Aggressiveness Traits in Colorectal Cancer Cells

Pérez-Moreno et al. found that phosphorylation of endothelin-converting enzyme-1c (ECE1c) at serines 18 and 20 by protein kinase CK2 promotes aggressiveness in colorectal cancer cells. This finding proposed a novel role of phospho-ECE1c as a biomarker for poor prognosis. CK2 is suggested to promote the malignant progression of colon cancer *via* increased stability of ECE1c protein (1). The inhibition of CK2 may be a potential target for the treatment of colorectal cancer.

## LINC00337 Regulates KLF5 and Maintains Stem-Cell Like Traits of Cervical Cancer Cells by Modulating miR-145

Han et al. identified that a long non-coding RNA (lncRNA) LINC00337 knockdown decreased the cancer stem cell (CSC)-like properties of CD44^+^/CD24^low/-^ sphere-forming cells. The results further demonstrate that LINC00337 regulates Kruppel-like factor 5 (KLF5) by sharing the binding sites with a microRNA, miR-145-5p, and highlighted the role of LINC00337 as an oncogenic lncRNA in cervical cancer. The miR-145 has predicted binding sequences on Oct4, Nanog, and Sox2, which induces cervical tumor-sphere differentiation, and inhibits invasion of the cervical CSCs (2). The regulation of the RNA network in cancer and stem cells is one of the important mechanisms in CSCs (3, 4).

## *STIL* Endows Oncogenic and Stem-Like Attributes to Colorectal Cancer Plausibly by Shh and Wnt Signaling

Pradhan et al. revealed that SCL/TAL1 interrupting locus (STIL) regulates phosphorylation of AKT and β-catenin expression in colorectal cancer. Sonic hedgehog (Shh) and Wnt signaling were highlighted in STIL-mediated regulation of CSC signatures. The role of *STIL* in stemness and drug resistance has been investigated, which found that the *STIL* regulates CSC markers CD133 and CD44, and drug-resistant markers, thymidylate synthase, ATP binding cassette subfamily B member 1 (ABCB1), and ABCG2. The Hedgehog (Hh)/Gli signaling and Wnt/β-catenin pathways cross-talks in colon cancer (5). The signaling pathways including Shh and Wnt signaling pathways are regulated in epithelial-mesenchymal transition and CSCs (6). The Shh-independent mechanism of STIL-induced up-regulation of CSC markers may be one of the interesting topics for future investigation.

## Tumor Growth in the High Frequency Medulloblastoma Mouse Model Ptch1^+/-^/Tis21^KO^ Has a Specific Activation Signature of the PI3K/AKT/mTOR Pathway and Is Counteracted by the PI3K Inhibitor MEN1611

Ceccarelli et al. found that a mouse model (*Ptch1^+/-^/Tis21^KO^*), which shows a significantly higher medulloblastoma frequency, demonstrated the activation signature of the phosphatidylinositol 3-kinase (PI3K)/AKT/mammalian target of rapamycin (mTOR) pathway. Patched (Ptch), a Hh receptor, inhibits the activity of Smoothened (Smo), a G protein (heterotrimeric guanosine triphosphate-binding protein)-coupled receptor (GPCR)-like molecule, which is a mainstream of the Hh pathway (7, 8). The activation of the PI3K pathway is involved in the resistance to a Smo antagonist in medulloblastoma (7). The administration of MEN1611, a PI3K inhibitor, demonstrated significant tumor growth inhibition relative to vehicle administration in the *Ptch1^+/-^/Tis21^KO^* model mice. Dual targeting of the Shh and PI3K/AKT/mTOR pathway would be one of the potential treatments for medulloblastoma.

## TGF-β1 Induces Immune Escape by Enhancing PD-1 and CTLA-4 Expression on T Lymphocytes in Hepatocellular Carcinoma

Bao et al. investigated the hepatocellular carcinoma microenvironment and the immune cell involvement and revealed that transforming growth factor β1 (TGF-β1) attenuated the immune function of T lymphocytes *via* the enhancement of the expression of programmed cell death 1 (PD-1) and cytotoxic T lymphocyte-associated antigen 4 (CTLA-4) on the T cells. TGF-β induces the calcineurin (CaN) pathway in cardiac myofibroblasts (9). The results demonstrated that TGF-β1 increased the activity of CaN and translocation of the nuclear factor of activated T cells 1 (NFATc1) into the nucleus, leading to the binding of the NFATc1 to PD-1 and CTLA-4 promoters and increased transcription of PD-1 and CTLA-4.

## Author Contributions

The author confirms being the sole contributor of this work and has approved it for publication.

## Funding

This work was supported by JSPS KAKENHI Grant Number JP21K12133, Japan Agency for Medical Research and Development (AMED), Grant Number JP21mk0101216, and Ministry of Health, Labour, and Welfare (MHLW), Japan.

## Conflict of Interest

The author declares that the research was conducted in the absence of any commercial or financial relationships that could be construed as a potential conflict of interest.

## Publisher’s Note

All claims expressed in this article are solely those of the authors and do not necessarily represent those of their affiliated organizations, or those of the publisher, the editors and the reviewers. Any product that may be evaluated in this article, or claim that may be made by its manufacturer, is not guaranteed or endorsed by the publisher.
